# LAD Occlusion From Alcohol Reflux During Alcohol Septal Ablation

**DOI:** 10.1016/j.jaccas.2024.103103

**Published:** 2025-02-05

**Authors:** Brian Kelley, Michael Halista, Thelsa Weickert, John Vavalle, Michael Yeung

**Affiliations:** Division of Cardiology, University of North Carolina, Chapel Hill, North Carolina, USA

**Keywords:** alcohol septal ablation, coronary occlusion, hypertrophic cardiomyopathy, no-reflow phenomenon

## Abstract

We present a case of alcohol septal ablation complicated by alcohol reflux with transient occlusion of the left anterior descending artery. This report discusses the main steps to the procedure and its complications, including the differential diagnosis and management.

## Graphical Abstract

**Figure:**
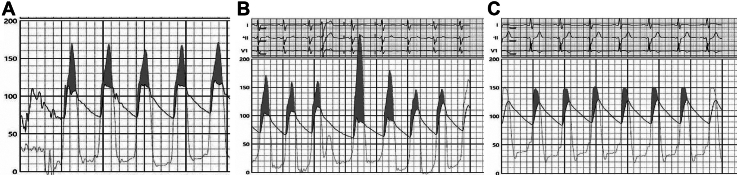

## History of Presentation

A 54-year-old man with a history of hypertrophic cardiomyopathy (status post–dual-chamber implantable cardioverter-defibrillator), hypertension, and obesity presents to the cardiac catheterization laboratory for elective alcohol septal ablation (ASA) caused by ongoing NYHA functional class III symptoms despite optimal medical therapy.

**Take-Home Messages**

• To understand the potential complications of alcohol septal ablation so prompt recognition and appropriate management can be pursued
• LAD occlusion caused by alcohol reflux is a rare but important complication of ASA that can be successfully managed via the administration of intracoronary saline and vasodilators

**Figure 1:**
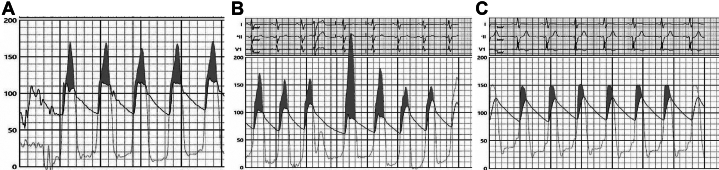
Measurement of the LVOT Gradient (A) Preablation resting gradient of approximately 50-60 mm Hg. (B) Preablation post-PVC gradient of approximately 110-120 mm Hg. (C) Postablation gradient of ∼20-25 mm Hg.

**Figure 2:**
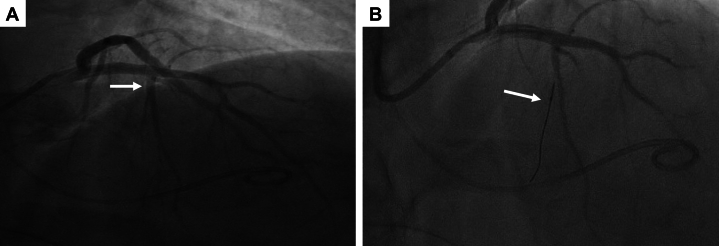
Identification of the Septal Perforator (A) Angiography shows a large-caliber, branching first septal perforator (arrow). (B) An interventional wire and over-the-wire balloon were advanced into the smaller branch of the first septal perforator.

During the initial portion of the procedure, the patient was resting comfortably and without any significant complaints. Double arterial access was obtained with 6-F sheaths placed in the right ulnar artery and left radial artery. A 6-F Langston pigtail catheter was advanced across the aortic valve into the left ventricular (LV) cavity for continuous monitoring of simultaneous LV-Ao pressure measurements. Severe left ventricular outflow tract (LVOT) obstruction was confirmed with a resting LVOT gradient of approximately 60 mm Hg and post-PVC gradient of up to 120 mm Hg (Figures 1A and 1B). Using the other arterial access, a 6-F EBU 3.5 guide catheter was used for engagement of the left coronary artery. Selective coronary angiography showed a patent left anterior descending artery (LAD) with a first septal perforator which was a large-caliber, branching vessel (Figure 2A). A 1.5 × 6.0 mm Sprinter over-the-wire (OTW) balloon was subsequently advanced over a 0.014-inch run-through wire into the smaller branch of the first septal perforator (Figure 2B). Upon removal of the wire, the balloon was inflated and confirmed to be well apposed on septal angiography by administering an injection of contrast through the central lumen of the balloon which did not show evidence of reflux. Before the injection of ethanol, we also performed myocardial contrast echocardiography to confirm proper localization of the interventricular septum supplied by the septal branch (Video 1). We then slowly infused a total of 1.5 mL of 99% dehydrated ethanol. Over the ensuing 10 to 15 minutes, he developed progressive chest pain with associated diaphoresis and malaise. During this timeframe, he had also developed progressive ST-segment elevation in the anterior leads, anterior wall motion abnormalities with reduced left ventricular ejection fraction on bedside echocardiogram (Video 2), and both his LV and Ao pressures dropped by approximately 100 mm Hg. Given this abrupt change in clinical presentation, the OTW balloon was deflated and retracted to perform angiography which showed complete occlusion of the mid LAD (Video 3).

Due to suspicion that his LAD occlusion was secondary to alcohol reflux, we immediately began administering serial flushes of intracoronary saline and adenosine. Quick-Cross and PriorityOne catheters were advanced into the LAD to provide better intracoronary delivery of these agents. While this was undertaken, the patient developed recurrent episodes of ventricular tachycardia/ventricular fibrillation which required multiple defibrillations from both an external defibrillator as well as his implantable cardioverter-defibrillator device. Although flow down the LAD remained sluggish, there was gradual improvement following aggressive flushing into the LAD (Video 4). After an additional 45 to 60 minutes, repeat angiography showed near resolution of the LAD occlusion (Video 5). His resting LVOT gradient had improved to 20 to 25 mm Hg (Figure 1C).

Once he had remained hemodynamically stable for a period of time, the procedure was concluded, and he was admitted to the cardiac intensive care unit for postoperative monitoring. Although still with some evidence of anterior hypokinesis, postoperative echocardiogram showed recovery of his left ventricular ejection fraction, improvement in the intracavitary gradient to approximately 23 mm Hg, and no evidence of a ventricular septal defect (Video 6). Additionally, his symptoms resolved and there was no recurrence of significant arrhythmias. He was discharged home 3 days after the procedure.

## Discussion

ASA represents a method of septal reduction therapy for symptomatic adults with obstructive hypertrophic cardiomyopathy who are at prohibitive surgical risk for septal myectomy with a Class IC recommendation per the 2020 AHA/ACC guidelines.^1^ First reported in 1994, the procedure is performed by administering 95% to 99% dehydrated ethanol into a suitable septal perforator artery to induce an iatrogenic myocardial infarction of the basal interventricular septum because of the cytotoxic effect of alcohol.^2^ Complications of the ASA procedure include complete heart block, ventricular arrhythmias, ventricular septal defect, and vessel injury. Although it is known to be a potential complication, LAD occlusion from alcohol reflux is uncommon and has been rarely described in the literature.^3^ Unsurprisingly, this can have devastating consequences the longer it goes unnoticed, such as anterior STEMI with ventricular tachycardia/ventricular fibrillation arrest as exemplified in our case.

**Figure 3:**
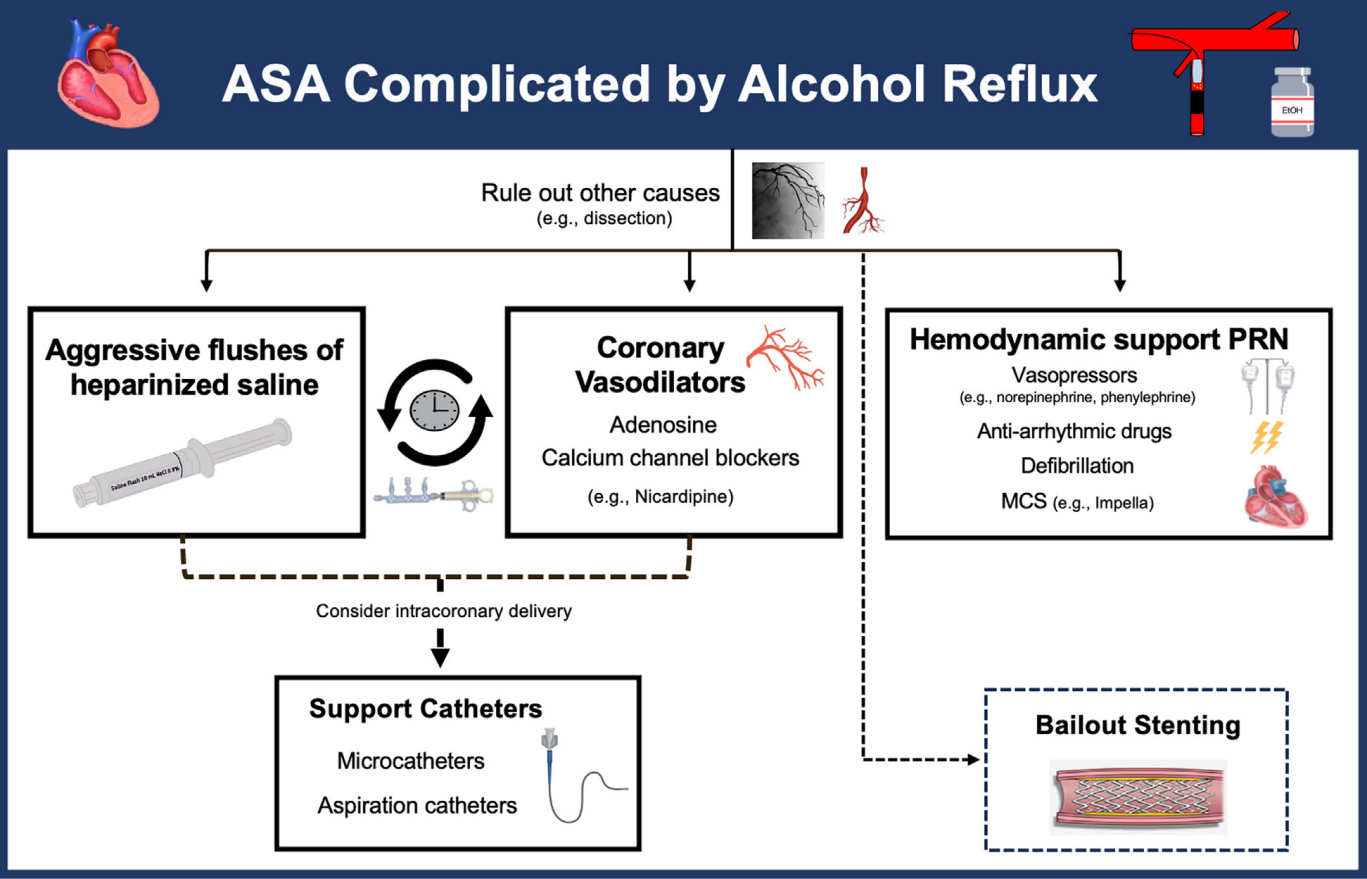
Proposed Algorithm for the Management of Alcohol Leakage During ASA ASA = alcohol septal ablation; MCS = mechanical circulatory support; PRN = as needed.

Alcohol reflux was suspected to be related to subtle movement of the guide catheter and balloon because of deep respirations intermittently taken by our patient during the procedure. The reflux of alcohol subsequently led to severe occlusion of the LAD segment just after the first septal perforator. Other important etiologies to consider on the differential diagnosis of LAD occlusion in this setting include dissection and vasospasm. These causes can also coexist, given there likely being some degree of alcohol-induced vasospasm. Although preventative strategies such as the meticulous monitoring of the OTW balloon during the instillation of alcohol into the septal perforator are obviously key, complications remain an inherent part of the various procedures we perform, and it is prudent for providers to have a plan of attack. Management of alcohol reflux during ASA is not well defined without evidence-based recommendations in current guidelines. Although stenting has been described as a way to manage LAD occlusion during ASA, we opted to defer placement of a stent given the lack of a mechanical cause of disrupted flow in the vessel.^3^ In our case, we viewed the best next step in management as being relatively similar to that of the *no-reflow* phenomenon with the use of intracoronary vasodilators (eg, adenosine, nicardipine, verapamil). Maybe of even greater importance was the repeated administration of heparinized saline flushes we performed to help “wash out” the alcohol from the vessel. Given the improvement in flow following aggressive intracoronary flushing, stenting was not felt to be necessary in our patient. Instead, stenting should be more so reserved as a bailout strategy or as an option for other causes of vessel occlusion such as dissection. Given the suspected reason for reflux being related to the patient’s deep breathing, we recommend a femoral approach to be utilized for the guide catheter to allow for better engagement and positioning in the aortic root. Based on our experience with this case, we propose an algorithm for the management of ASA complicated by alcohol reflux (Figure 3).

## Conclusions

LAD occlusion secondary to alcohol reflux is a known but infrequently described complication of ASA. Prompt recognition and management is essential to abort the ensuing severe, irreversible damage to the myocardium. To the best of our knowledge, this case report highlights this complication and its first successful management via aggressive flushing of the LAD using intracoronary saline and adenosine.

## Funding Support and Author Disclosures

The authors have reported that they have no relationships relevant to the contents of this paper to disclose.

## Appendix

Video 1 MCE Performed Intraoperatively to Help Identify Area of the Interventricular Septum Supplied by the First Septal Perforator

Video 2 Bedside Echocardiogram Shows Interval Development of Anterior Wall Motion Abnormalities and Reduced LVEF

Video 3 Coronary Angiogram Shows Complete Occlusion of the LAD

Video 4 Coronary Angiogram Shows Gradual Improvement of Flow in the LAD Following Aggressive Flushing

Video 5 Coronary Angiogram at the End of the Case Shows Near Resolution of Flow in the LAD

Video 6 Postoperative Echocardiogram Shows Recovery of His LVEF With Improvement in the Intracavitary Gradient to Approximately 23 mm Hg and No Evidence of a VSD
